# Hong Kong Chinese adults’ knowledge of exercise recommendations and attitudes towards exercise

**DOI:** 10.3399/bjgpopen17X100929

**Published:** 2017-05-03

**Authors:** Man Kin Wong, Sai Yip Ronald Cheng, Tsun Kit Chu, Cheuk Nang Lee, Jun Liang

**Affiliations:** 1 Associate Consultant, Department of Family Medicine and Primary Health Care, Tuen Mun Hospital, New Territories, Hong Kong SAR, China; 2 Associate Consultant, Department of Family Medicine and Primary Health Care, Tuen Mun Hospital, New Territories, Hong Kong SAR, China; 3 Associate Consultant, Department of Family Medicine and Primary Health Care, Tuen Mun Hospital, New Territories, Hong Kong SAR, China; 4 Research Assistant, Department of Family Medicine and Primary Health Care, Tuen Mun Hospital, New Territories, Hong Kong SAR, China; 5 Chief of Service, Department of Family Medicine and Primary Health Care, Tuen Mun Hospital, New Territories, Hong Kong SAR, China

**Keywords:** knowledge on exercise guidelines, traditional and lifestyle physical activities, attitudes towards exercise

## Abstract

**Background:**

Physical inactivity is known to be one of the major risk factors for many chronic conditions.

**Aim:**

To determine Hong Kong Chinese adults’ physical activity (PA) knowledge, its relationship with certain variables including sex, education, age, and its correlation with different types of chronic diseases, such as heart disease, cerebrovascular disease, diabetes mellitus, obesity, and others. The Hong Kong Chinese adults' general attitudes towards PA will also be examined.

**Design & setting:**

Cross-sectional study in one primary care centre.

**Method:**

A randomized sample of Chinese adults aged >18 years with anonymous self-administered questionnaires.

**Results:**

The mean percentage of correct responses for exercise guidelines was 62.3%, 84.5% for traditional PA, and 48.4% for lifestyle PA, respectively. Traditional PA refers to exercises which use large muscle groups. Lifestyle PAs include activities which can be done throughout the day. The total mean percentage of correct responses was 67% (knowledge score 13.4 +/– 3.34). There were no significant differences between PA knowledge and sex, education levels, age groups, and presence of chronic diseases (*P*>0.05), but the knowledge level for lifestyle PA was less than that of traditional PA (*P*<0.001). A weak correlation was found between responders’ activeness for a health benefit and the PA knowledge levels (*P*>0.05). Two hundred and sixy-six (93.3%) responders reported a willingness to maintain or start exercise.

**Conclusion:**

The results suggest a need for more education about the latest PA recommendations, especially lifestyle PA. The weak correlation between PA knowledge and actual behaviour showed that PA knowledge itself might not affect PA behaviour. The enhancement of the general public’s knowledge, motivation, and psychosocial support along with stage-of-change interventions and the provision of counselling skills may result in PA behaviour change, which in turn can lead to the achievement of health benefits.

## How this fits in

Local data on the knowledge of exercise guidelines, traditional and lifestyle PA are lacking. Given the busy lifestyle of people in Hong Kong, this study may have important implications for healthcare providers, enabling them to prescribePA suitable for different lifestyles, and based on updated exercise guidelines.

## Introduction

Physical inactivity is well known to be one of the major risk factors for heart disease, cerebrovascular disease, diabetes mellitus, hypertension, some types of cancers, and obesity in both males and females of any age.^1^

In Hong Kong, the Behavioural Risk Factor Survey conducted by the Department of Health in April 2014 revealed that about one-third (33.7%) of adults aged 18–64 years had not done any moderate or vigorous PA for at least 10 minutes at a time and less than two-fifths (39.8%) had done some vigorous PA during the week prior to the survey. This level of PA was clearly not enough for optimal health gain.^2^

According to the findings of the Consultancy Study of Sports for All, the primary reasons for not participating in PA include 'no spare time due to work or study' (30.7%), 'tiredness' (17.5%), and 'laziness' (14.6%).^3^

An understanding of the public’s PA knowledge is important^4,5^ because this could help in developing health promotion and PA interventions. Previous local studies on PA have mainly focused on the knowledge of how PA impacts general health. Overseas studies^6^ have assessed populations’ knowledge on exercise recommendations, but locally, there is a dearth of such data.

The PA guidelines sufficient to provide a health benefit suggest moderate and/or vigorous PA. The recommendations state that moderate activity for 30 minutes per day for a minimum of 5 days per week should be undertaken.^7^ The 30 minutes per day can be accumulated over three sessions. Vigorous PA can be done 20 minutes per day for a minimum of 3 days per week.^7^ It has now been established that traditional types of PA and lifestyle PAs benefit health. Traditional PAs use large muscle groups and include activities such as jogging, aerobic exercises, cycling, dancing, recreational sports (such as team and individual sports), and include swimming, walking, and weight lifting. Lifestyle PAs are activities that can be undertaken throughout one's day. Typically, moderate PAs are perceived as those >3 metabolic equivalent of tasks (METs) while vigorous PAs are >6 METs.^8^

The aim of this study was to determine the knowledge of adults in Hong Kong on exercise guidelines, traditional and lifestyle PA, and their attitude towards exercise. Items based on the Centers for Disease Control and Prevention/American College of Sports Medicine principles included knowledge of exercise guidelines, and traditional and lifestyle PA.

## Method

This was a cross-sectional study carried out in one primary care family medicine centre (Madam Yung Fung Shee Health Centre) in Hong Kong. The sampling frame for this study was chosen via convenient sampling. A random sample of 285 Chinese adults aged ≥18 years attending the clinic were invited to participate in this study from May to June 2015. Exclusion criteria included those aged <18 years or those not be able to give consent. A structured questionnaire was designed for this study as there had been no validated Chinese questionnaire to assess the level of knowledge on exercise guidelines, and traditional and lifestyle PA prior to this study. There were 20 questions to assess responders' knowledge of exercise guidelines, traditional and lifestyle PA, along with attitude towards exercise (details available from author on request). All questions were originated from a validated questionnaire in assessment of PA knowledge among US citizens.^6^ The number of correct responses to these questions formed a knowledge score for each individual. Attitude towards exercise was assessed by two questions on responders’ perceived sufficiency of exercise for a health benefit, and willingness to maintain or start exercise to achieve a health benefit.

A focus group was held among patients of different educational levels. The researchers also piloted the first few cases. The questionnaires were understandable and no major corrections were needed.

An anonymous self-administered questionnaire written in Chinese was distributed to each responder by a supporting staff member who explained the purpose of the study, its voluntary nature, and confidentiality of data. The principal investigator provided on-site explanation for any responder who had difficulties in understanding the questionnaire. Responders gave their questionnaires to their GPs who also acted as interviewers where further clarification was needed to assist completion of the questionnaires. GPs also double-checked the information provided through the Clinical Management System provided by the Hospital Authority in Hong Kong.

The questionnaires included open questions and multiple choice. The choices of response were 'True/Yes', 'False/No', and 'do not know'. The responders scored 1 point for each correct response and zero for an incorrect or 'do not know' response. The responder’s score out of 20 was their degree of awareness of the effects of PA on one’s health.

Sample size estimation is based on the total score average (76.8%) which was the percentage of responders correctly answering individual items according to the study by Morrow and colleagues.^6^

Using the desired precision as 0.05, the estimated sample size was 246. The following formula was used:

$$n=\frac{Z^{2}1-\alpha /2\;P(1-P)}{d^{2}}$$

where *n* was the estimated sample size, P was the estimated proportion, d was the desired precision and α was set at 0.05 level. Results were reported as the percentage of responders who correctly responded to each item. Additionally, a mean percentage had been determined for each of the three subscales by dividing the individual’s PA guidelines and traditional PA scores by 8 (the number of traditional PAs) and 6 (for lifestyle PAs). Details on demographic variables like sex, education level, age, and the presence of chronic diseases had been collected to determine if the total knowledge was a function of them. Education level was coded as completed primary school or below/completed secondary school/completed university (tertiary) or above. Age was coded as 18–35/36–60/≥61 years.

Chronic diseases only included those with cardiorespiratory risk (for example, hypertension, diabetes, ischaemic heart disease, chronic obstructive airway disease, or asthma) or musculoskeletal diseases, such as osteoarthritis of the knee. Responders were coded as conducting sufficient or insufficient PA to achieve a health benefit to be able to determine any differences in their knowledge level. Statistical analyses were done using the Statistical Package for Social Sciences (SPSS).

## Results

Two hundred and ninety-three questionnaires were collected while 306 questionnaires were given out. Thirteen were refused. The response rate was 95.8%. Valid questionnaires for analysis were 285 as eight were excluded because of incompleteness of the information. Detailed demographic characteristics of responders are shown in Table 1. This questionnaire achieved high internal consistency and reliability (Cronbach’s α 0.856).

**Table 1.** Descriptive statistic of responders (*n* = 285)

Variables	Male	Female
*n*		
**Age, years**		
18–35	15	16
36–60	82	83
≥61	42	45
**Education**		
Primary or below	28	51
Secondary	100	80
Tertiary or above	11	13
**Chronic illness**		
Yes	100	94
No	39	50

Sample sizes do not total 285 because of missing data.

The highest score among responders was 18. The percentage of responders correctly answering individual items varied from 34.4 (furniture moving) to 95.4 (walking). The mean percentage of correct responses for exercise guidelines was 62.3%; 84.5% for traditional PA and 48.4% for lifestyle PA, respectively. The total mean percentage of correct responses was 67.0% (knowledge score 13.4 +/– 3.34), which indicated that the responders could correctly answer more than half of the questions. Detailed results are shown in Table 2.

**Table 2.** Overall knowledge about Centers for Disease Control and Prevention/American College of Sports Medicine PA guidelines (*n* = 285)

**PA guidelines**		**% correct**
	Minimum days per week	53.3
	Minimum minutes per day	65.6
	Vigorous activity needed to achieve a health benefit	79.9
	Moderate activity is beneficial	65.3
	Three 10-minute bouts of exercise	35.2
	Minimum of 30 minutes per day	74.6
	Mean per cent correct for 6 items	62.3
**Traditional PA**		
	Aerobic class	84.2
	Cycling	91.6
	Dancing	79.3
	Jogging/running	90.9
	Recreational sports	86.9
	Swimming	91.2
	Walking	95.4
	Weight lifting	56.3
	Mean per cent correct for 8 items	84.5
**Lifestyle PA**		
	Household cleaning	56.2
	Furniture moving	34.4
	Gardening and lawn work	53.7
	Preparing meals	42.3
	Playing a musical instrument	41.5
	Raking leaves	62.5
	Mean per cent correct for 6 items	48.4
**Mean per cent correct for 20 items**		67.0

PA = physical activity.

The proportions of three choices of response in each question, as shown in Table 3, indicate a few questions that need attention. Only 35.2% of responders knew that one continuous session and multiple shorter sessions (of at least 10 minutes) are both acceptable to accumulate the desired amount of daily exercise. More than one-third of the responders (43.7%) did not know that weight lifting, a form of resistance exercise, can provide health benefits (item 7n). More than half of responders demonstrated limited knowledge on the effects of lifestyle exercises on health (items 7g–i).

**Table 3.** Proportions of different answers in each PA knowledge question

**Questions**	**Answers**				
	**Correct, *n* (%)**	**Incorrect, *n* (%)**			
1. What is the minimum number of days per week you believe a person must be physically active in order to receive any health benefit? (3,4,5)	152 (53.3)	133 (46.7)			
2. What is the minimum length of time (in minutes) one needs to be physically active throughout a typical day in order to achieve a health benefit? (30 minutes)	187 (65.6)	98 (34.4)			
	**Agree, *n* (%)**	**Disagree, *n* (%)**		**Don’t know, *n* (%)**	
3. Vigorous levels of physical activity are necessary to provide a health benefit (F)	15 (5.3)	227 (79.6)		42 (14.7)	
4. Moderate levels of physical activity do NOT provide any health benefits (F)	50 (17.5)	186 (65.3)		49 (17.2)	
5. Ten minutes of physical activity three times per day provide the same health benefits as a single session of 30 minutes (T)	100 (35.1)	103 (36.1)		81 (28.4)	
6. Everyone should get 30 minutes of moderate physical activity most days of the week (T)	212 (74.4)	19 (6.7)		53 (18.6)	
7. Which of the following physical activities in general do you believe will provide a health benefit?					
a. Aerobic class (T)	240 (84.2)	8 (2.8)		37 (13)	
b. Biking (T)	261 (91.6)	10 (3.5)		14 (4.9)	
c. Dancing (T)	226 (79.3)	16 (5.6)		43 (15.1)	
d. Gardening and lawn work (T)	151 (53.0)	60 (21.1)		70 (24.6)	
e. Household cleaning (T)	159 (55.8)	76 (26.7)		48 (16.8)	
f. Jogging/running (T)	259 (90.9)	10 (3.5)		16 (5.6)	
g. Playing a musical instrument (F)	68 (23.9)	118 (41.4)		98 (34.4)	
h. Moving furniture (T)	98 (34.4)	129 (45.3)		58 (20.4)	
i. Preparing meals (F)	102 (35.8)	120 (42.1)		62 (21.8)	
j. Ranking leaves (T)	178 (62.5)	55 (19.3)		52 (18.2)	
k. Recreational sports (such as team and individual sports) (T)	245 (86.0)	10 (3.5)		27 (9.5)	
l. Swimming (T)	259 (90.9)	14 (4.9)		11 (3.9)	
m. Walking (T)	270 (94.7)	5 (1.8)		8 (2.8)	
n. Weight lifting (T)	160 (56.1)	58 (20.4)		66 (23.2)	
**Attitude**	**Not nearly enough, *n* (%)**	**Not enough, *n *(%)**	**Don’t know, *n* (%)**	**Enough, *n* (%)**	**More than enough,** *n* **(%)**
Do you think that you have a sufficient level of activity to achieve a health benefit?	22 (7.7)S	61 (21.4)	34 (11.9)	98 (34.4)	69 (24.2)
	**Agree, *n* (%)**	**Disagree, *n* (%)**		**Don’t know, *n* (%)**	
Do you think that you are willing to begin or maintain exercise for a health benefit?	266 (93.3)	2 (0.7)		17 (6)	

^a^T = true. F = false. ^b^Sample size does not total 285 because of no responses to item.

Tables 4 and 5 showed the various average percentages of correct answers and mean knowledge score with respect to different demographic and disease factors respectively. There were no significant differences between PA knowledge and sex, education levels, age groups, and presence of chronic diseases (*P*>0.05).

**Table 4.** Knowledge of Centers for Disease Control and Prevention/American College of Sports Medicine PA guidelines by demographic groups

	**Total^a^**	**Physical** **activity guidelines^a^**	**Traditional** **physical** **activities^a^**	**Lifestyle physical activities^a^**
**Sex**				
Male (*n* = 140)	68.5	63.9	85.1	50.8
Female (*n* = 145)	65.2	60.6	83.4	45.6
**Education level^b^**				
Primary or below (*n* = 79)	65.4	57.0	81.8	52.1
Secondary (*n* = 180)	66.6	63.6	84.6	45.7
Tertiary or above (*n* = 24)	71.6	68.1	88.5	52.8
**Age, years**				
18–35 (*n* = 31)	65.5	63.4	84.3	42.5
36–60 (*n* = 167)	67.3	62.4	85.5	48.0
≥61 (*n* = 87)	66.3	61.5	81.8	50.6

^a^Average % correct. ^b^Sample size does not total 285 because of no responses to item.

**Table 5.** PA knowledge score according to different demographic, disease factors and willingness

	*n*	P**hysical activity knowledge score, mean (SD)**	***P*-value**
**Sex**			0.099
Male	140	13.69 (3.200)	
Female	145	13.04 (3.444)	
**Education^a^**			0.277
Primary or below	79	13.09 (3.603)	
Secondary	180	13.33 (3.204)	
Tertiary or above	24	14.33 (3.371)	
**Age, years**			0.813
18–35	31	13.10 (3.124)	
36–60	167	13.46 (3.008)	
≥61	87	13.26 (3.981)	
**Chronic illness**			0.131
Yes	195	13.56 (3.227)	
No	90	12.92 (3.542)	
**Willingness**			<0.050
Yes	266	12.17 (3.128)	
No	19	8.11 (5.248)	

^a^Sample size does not total 285 because of no responses to item. SD = standard deviation.

The knowledge level for lifestyle PA was less than that of the traditional PA (*P*<0.001) overall, in which the mean knowledge score for lifestyle PA was 2.89 +/– 1.49 while for traditional PA it was 6.74 +/– 1.73.

Finally, from Table 6 a weak correlation was found between responders’ activeness for a health benefit and the PA knowledge levels (*P*>0.05).

**Table 6.** Pearson correlations between perceived sufficiency of exercise and knowledge of physical activities

**Correlations**		
		**Sufficient**
Sufficient	Pearson Correlation	1
	*P*-value	–
	*n*	285
Traditional	Pearson Correlation	0.046
	*P*-value	0.434
	*n*	285
Lifestyle	Pearson Correlation	–0.009
	*P*-value	0.878
	*n*	285
Physical guidance	Pearson Correlation	–0.018
	*P*-value	0.756
	*n*	285
Total knowledge	Pearson Correlation	0.012
	*P*-value	0.835
	*n*	285

Mean sufficient score was 3.46 +/– 1.278.

Two hundred and sixty-six (93.3%) responders reported willingness to maintain or start exercise. Those willing to begin or maintain exercise had a significantly higher PA knowledge than others (Table 5).

## Discussion

### Summary

More education about the latest PA recommendations, especially lifestyle PA is needed. The weak correlation between PA knowledge and actual behaviour showed that knowledge itself might not affect behaviour. Enhancement of the public’s knowledge, motivation, and psychosocial support along with stage-of-change interventions and proper counselling skills, for example, motivational interviewing, may result in PA behaviour change to achieve health benefits.

### Strengths and limitations

This study was a cross-sectional design but not a controlled trial, and was thus unable to provide causal relationship as the researchers only tested the association between variables. Generalisability was limited because the data were collected from only a single clinic with convenient sampling. The modest sample size may limit the applicability of the studies. It contained self-reported data which tended to have recall bias. The use of subjective measures of PA may be fraught with overestimation or problems with recall.^9^ Data on income, which are likely to be influential factors on doing exercise, were not measured. Not all professions were assessed in this study; for example, those who were physically active as part of their job may bias the study findings. The high frequency of results for willingness to begin or maintain PA might be explained by possible interviewer bias towards those handing in their questionnaires. Finally, the validity of the questionnaire has yet to be determined.

Despite these limitations, this study is one of the first to assess PA knowledge of the exercise recommendations in an adult Hong Kong Chinese population. It found that their PA knowledge was generally lower than previously known. In addition, their knowledge of lifestyle PA was lower than that of traditional PA.

### Comparison with existing literature

The average mean knowledge scores were generally lower than that from studies by Morrow and colleagues^6^ in which 76.8% (knowledge score 16 +/– 2.2) were correctly answered. A nationwide random-digit dialing was undertaken to arrive at a representative sample of US adults. For an approximate 1-week period, it resulted in 2002 responders after three telephone attempts. The increased number of responders may be the reason accounting for the difference between the two studies.

The results revealed the responders in this study had knowledge about how to be physically active for a health benefit that varied by PA type. This is illustrated in Table 2, where percentages of correct responses are presented by subscales and across all 20 items. Table 4 presents similar results by demographic variables. However, knowledge on different subscales is generally lower than Morrow and colleagues's study,^6^ except for the minimum minutes per day and the vigorous activity necessary to achieve a health benefit. Only 56.3% of responders viewed weight lifting as an activity that can provide a health benefit. This is far below the results of Morrow and colleagues,^6^ in which 82.4% viewed it as a healthy activity. This may be related to the concept that some Chinese viewed weight lifting as a strenuous activity, which might therefore not contribute to a health benefit. In another study, excellent knowledge about recommended PA guidelines was seen^10^ which may be related to the closed-ended questions asked, while open-ended questions were asked about the recommended PA guidelines for an adult in this study and may have been too general for responders. Overall, the scores for the exercise guidelines suggest a need for further education about the latest PA recommendations. A lack of association between PA knowledge and various demographic or disease factors were shown in this study.

Education level was found to positively associate with PA knowledge in a local study for diabetic patients.^11^ Dishman^12^ and Sallis and colleagues^13^ also suggested increasing PA knowledge through education is an effective method of promoting PA. Although the result of this study is different, programmes for education and promotion of PA should be advocated for all ages, sex, and education, irrespective of the presence of a chronic illness. Furthermore, this study demonstrated that the sample population had more knowledge about traditional PAs than lifestyle PAs, consistent with the study by Morrow and colleagues.^6^

The current lifestyle behaviour activities and PA guidelines (for example, accumulation of 30 minutes of PA), were still not as well known by the public. It is possible that the responders had various perceptions of lifestyle activities. For example, actively playing with children is different from vigorously playing with children.^8^ Actively playing with children includes walking, running, or climbing, while vigorously playing with children includes running longer distances, or playing strenuous games with them. Actively playing with children is represented as a moderate type of PA and counts for at least 3 METs, while vigorous play accounts for at least 6 METs. To raise the awareness of PA, education programmes, such as physiotherapy, could incorporate PA guidelines and lifestyle PA knowledge into their programmes. Active transportation, such as climbing the stairs, could also be advocated as a way to increase lifestyle PA.^14^

### Implications for research and practice

The Pearson correlation results (Table 6) demonstrate a weak correlation between knowledge of physical activity for a health benefit and actual PA undertaken. This finding is consistent with those previous studies that showed a lack of a direct association between PA knowledge and reported weekly duration of PA.^15–16^

One of the previous studies suggested that the poor relationship between knowledge and behaviour might result from an insufficient assessment of knowledge.^15^ Thus, a definite and objective system is needed to measure PA knowledge. The researchers believe that the 20-item questionnaire is a concise and effective tool. The various factors and barriers leading to PA could be evaluated by further qualitative research.

Most of the responders reported sufficiency in performing exercises and willingness to start or maintain exercise. It has been reported that attitude to exercise and knowledge of exercise are weakly related to participation in PA. Non-availability of facilities or supportive environment may present important barriers in avoiding desired participation in PA.^17–19^ Thus, the establishment of a better environment for sports is important.

Nevertheless, to enhance a positive attitude towards exercise, healthcare workers’ counselling skills on healthy lifestyle could be objectively assessed and further training provided where necessary. Motivational interviewing is one way achieving this.
